# The Cancer-Protective Potential of Protocatechuic Acid: A Narrative Review

**DOI:** 10.3390/molecules29071439

**Published:** 2024-03-23

**Authors:** Jorge Cadena-Iñiguez, Edelmiro Santiago-Osorio, Nancy Sánchez-Flores, Sandra Salazar-Aguilar, Ramón Marcos Soto-Hernández, María de la Luz Riviello-Flores, Víctor Manuel Macías-Zaragoza, Itzen Aguiñiga-Sánchez

**Affiliations:** 1 Postgraduate College, Campus San Luis Potosí, Salinas de Hidalgo, San Luis Potosí 78622, Mexico; jocadena@colpos.mx; 2 Hematopoiesis and Leukemia Laboratory, Research Unit on Cell Differentiation and Cancer, Faculty of High Studies Zaragoza, National Autonomous University of Mexico, Mexico City 09230, Mexico; edelmiro@unam.mx (E.S.-O.); nancy.ibra@gmail.com (N.S.-F.); 3 Specialized Equipment Laboratory, Faculty of High Studies Zaragoza, National Autonomous University of Mexico, Mexico City 09230, Mexico; saguilars@colpos.mx; 4 Postgraduate College, Campus Montecillo, Km. 36.5, Carretera México-Texcoco, Montecillo, Texcoco 56230, Mexico; msoto@colpos.mx (R.M.S.-H.); marluriviello@gmail.com (M.d.l.L.R.-F.); 5 Department of Biomedical Sciences, Faculty of Medicine, Faculty of Higher Studies Zaragoza, National Autonomous University of Mexico, Av. Guelatao 66, Iztapalapa, Mexico City 09230, Mexico; iram66671@yahoo.es

**Keywords:** antioxidant activity, protocatechuic acid, chemopreventive, anticancer activity

## Abstract

Cancer is one of the leading causes of death worldwide, making the search for alternatives for its control a critical issue. In this context, exploring alternatives from natural sources, such as certain vegetables containing a variety of secondary metabolites with beneficial effects on the body and that play a crucial role in the fight against cancer, is essential. Among the compounds with the greatest efficacy in controlling this disease, those with antioxidant activity, particularly phenolic com-pounds, stand out. A remarkable example of this group is protocatechuic acid (PCA), which has been the subject of various revealing research on its activities in different areas. These studies sustain that protocatechuic acid has anti-inflammatory, antimutagenic, antidiabetic, antiulcer, antiviral, antifibrogenic, antiallergic, neuroprotective, antibacterial, anticancer, antiosteoporotic, anti-aging, and analgesic properties, in addition to offering protection against metabolic syndrome and con-tributing to the preservation of hepatic, renal, and reproductive functionality. Therefore, this paper aims to review the biological activities of PCA, focusing on its anticancer potential and its in-volvement in the control of various molecular pathways involved in tumor development, sup-porting its option as a promising alternative for cancer treatment.

## 1. Introduction

Cancer is a leading cause of death and a major obstacle to prolonging life expectancy worldwide. According to estimates by the World Health Organization (WHO) in 2019, cancer is the first or second leading cause of death before the age of 70 in 112 out of 183 countries and third or fourth in 23 other countries [1].

The process of carcinogenesis, which is responsible for tumor development, is enhanced by multiple factors that manifest their widespread presence across a range of different cancer types in humans [2,3]. These fundamental characteristics, in which cells transition from a normal to a neoplastic growth, include the ability to drive proliferative signaling, bypass growth suppressive mechanisms, cell death resistance, obtain replicative immortality, induce angiogenesis, activate invasion and metastatic processes, reprogram cellular metabolism, and evade immune destruction [3]. This microenvironment promotes the accumulation of reactive oxygen species (ROS) can also alter the redox balance in the body and activate or inhibit signaling cascades (NF-kb, MAPKs, Keap1-Nrf2-ARE, and PI3K/Akt), causing alteration to ion channels and carrier molecules. These imbalances can cause inflammation and cell death leading to the development of pathologies such as cardiovascular disease, diabetes mellitus, neurodegenerative disorders, cancer and accelerated aging [4]. To prevent these diseases, antioxidant-rich or fortified therapies and diets could be effective. The latter emerge as promising tools to prevent or, at least, mitigate the functional deterioration of tissues and organs [5,6]. In this endeavor, natural sources such as fruits, vegetables, seeds, and spices play a crucial role due to their content of phytochemicals, particularly phenolic compounds [7]. These compounds have garnered significant attention due to their antioxidant, anti-inflammatory, and anticancer properties, which are considered key in protecting against various chronic disorders, including neurodegenerative diseases, diabetes, cardiovascular diseases, and cancer [8].

Among phenolic compounds, protocatechuic acid (PCA) may be a beneficial agent that can exert positive effects on critical stages of carcinogenesis due to its remarkable antioxidant activity, ability to block specific carcinogen binding sites to DNA, chemopreventive properties, normalization of cellular metabolism, and anti-inflammatory actions [9,10].

Therefore, this paper aims to present an update on the characteristics of protocatechuic acid (PCA), its bioavailability, its anticancer potential, and its involvement in the regulation of various molecular pathways, which could have relevant clinical applications in cancer prevention and even as an antineoplastic agent.

## 2. Characteristics and Sources of PCA

Natural polyphenols are secondary plant metabolites found in a variety of plant-based foods, with characteristics such as bitterness, astringency, color, flavor, odor, and oxidative stability of foods [11]. These valuable compounds are present in various parts of plants, such as fruits, vegetables, nuts, seeds, leaves, flours, roots, and barks [12].

Polyphenols are a large group of naturally occurring organic compounds characterized by one or more aromatic rings bonded to one or more hydroxyl groups in their structure [11,13]. Within these, dietary polyphenolic compounds envelop approximately 8000 variants and are categorized into four main subclasses noted for their ability to block carcinogenic processes and suppress cancer progression: flavonoids (such as flavanols, flavones, isoflavones, flavanones, anthocyanidins and flavanols), phenolic acids (derived from hydroxybenzoic acids, such as protocatechuic acid, and hydroxycinnamic acid, such as caffeic, ferulic, and coumaric acid), stilbenes, and lignans [13,14].

Phenolic acids constitute 30% of dietary polyphenols [14], including hydroxybenzoic acids such as protocatechuic acid (PCA), which, along with gallic, p-hydroxybenzoic, vanillic, ellagic, and syringic acids, can also be a product of the metabolism of anthocyanins and proanthocyanidins belonging to the flavonoid group. These acids presents in various forms, either as free acids, bounded to sugars or organic acids, or as structural components in more complex compounds, such as lignans and hydrolysable tannins [15].

**Figure 1:**
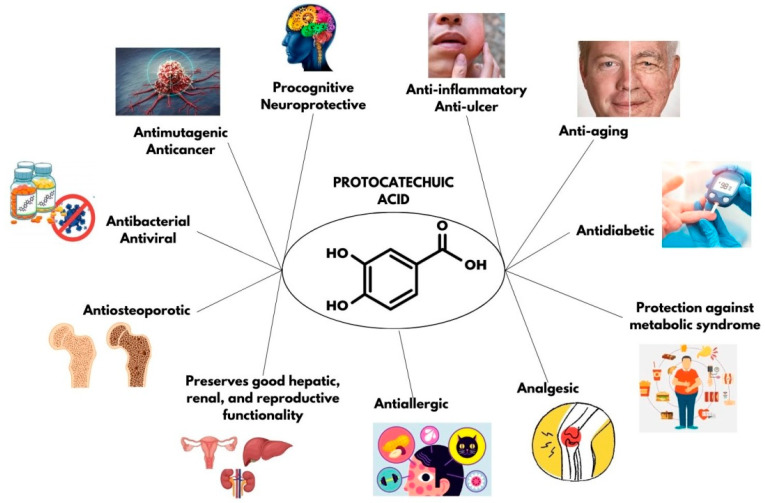
Chemical structure and properties of protocatechuic acid (3,4-dihydroxybenzoic acid).

In particular, protocatechuic acid, also known as 3,4-dihydroxybenzoic acid (Figure 1), is a natural phenolic compound present in a variety of foods, such as olives (*Olea europaea*), hibiscus (*Hibiscus sabdariffa*), and white wine grapes (*Vitis vinifera*) [16]. In addition to its recognized antioxidant activity, protocatechuic acid has been the subject of numerous studies both in vitro and in vivo, revealing a spectrum of multi-directional biological activities including anti-inflammatory, anti-mutagenic, anti-diabetic, anti-ulcer, antiviral, anti-fibrogenic, anti-allergenic, anti-allergic, procognitive, neuroprotective, antibacterial, anti-carcinogenic, anti-osteoporotic, anti-aging, and analgesic. In addition to its protective effects against metabolic syndrome, it also exhibits procognitive, neuroprotective, antibacterial, anticancer, anti-osteoporotic, anti-aging, and analgesic properties. Furthermore, PCA supports healthy liver, kidney, and reproductive function [5,17,18,19].

PCA, when extracted for study, appears in the form of a crystalline powder, with a color ranging from gray to brown [20]. Its molecular weight is 154.12 g/mol. It is soluble in ethanol and ether but slightly soluble in water (1:50). Its boiling point is 410 °C, and its melting point is 202–204 °C. Its density is 1.68 g/cm^3^. Despite its general stability, PCA may be incompatible with oxidizing agents and strong bases [9,17,18,20].

PCA quantity varies considerably depending on the type of plant food and is subject to the influence of several factors, such as the maturity level at harvest, the vegetation period, environmental conditions, cooking level, and storage conditions [16,21]. More than thirty plant-based foods containing PCA have been identified, with some, such as potatoes, Huajicor variety hibiscus, onions, or *Sechium compositum* (chayote 30 Gy SC- *S. compositum)*, notably rich in this substance, and others with lower concentrations such as amaranth, olive oil, and wheat (Table 1).

Lazcano-Peralta (2009) mentions that the nutritional composition of foods changes when subjected to cooking processes, as some of their components are heat-sensitive. The study examined various cooking methods on *Amaranthus hypochondriacus* L. leaves. After subjecting them to techniques such as blanching, sautéing, and frying, PCA could not be detected, as this compound degraded due to the high temperatures used in these processes. However, it was found that the steam blanching method was the most effective in preserving PCA to a greater extent, confirming that this compound shows higher concentrations in raw extracts compared to those subjected to heat [43]. Indeed, it is reported that the amount of protocatechuic acid (PCA) decreases dramatically when subjected to thermal treatments, reaching up to 90% loss after boiling at 100 °C [5,44].

## 3. PCA Absorption and Bioavailability

Several studies in humans have consistently shown that protocatechuic acid (PCA), generated in the gastrointestinal tract from the microbial catabolism of certain flavonoids (such as anthocyanins, procyanidins, and quercetin), promotes high bioavailability [45,46,47,48]. It is now recognized that the concentration of PCA in the human body can exceed the amount expected from direct consumption, since it becomes a main product of polyphenol metabolism, especially derived from anthocyanins and proanthocyanidins [49].

The absorption process of PCA derived from anthocyanins involves a sequence of complex and specific steps in the gastrointestinal system. Initially, PCA bioavailability begins with the digestion of food in the oral cavity, where tissues, saliva, and oral microbiota enhance intracellular absorption and phase II conversion of anthocyanins [50].

**Figure 2:**
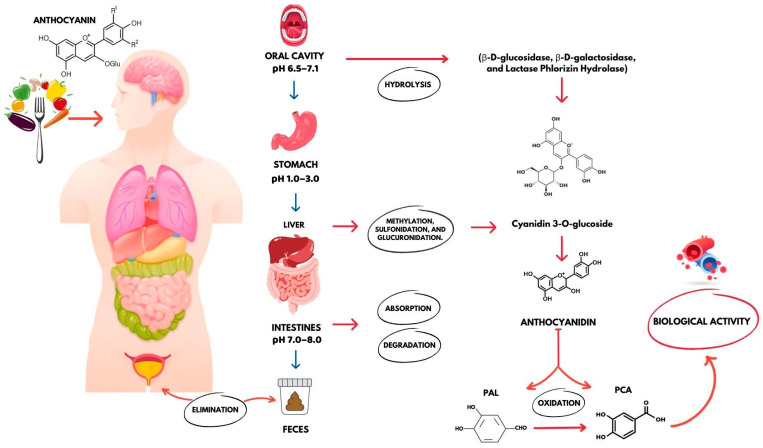
Absorption process of anthocyanin-derived PCA in the human body: absorption, distribution, metabolism, and elimination. The absorption of nutrients in the gastrointestinal tract is influenced by the pH of the gastrointestinal tract, which varies significantly between different sectors, ranging from 1.0 to 8.0. Once in the blood, protocatechuic acid (PCA) demonstrates not only antioxidant activity, but also various health-promoting properties, such as anticancer, anti-inflammatory, disease protection, and organ enhancement.

Through the deglycolization of anthocyanins (Figure 2), enzymes present in the stratified squamous surface epithelium and terminal ducts of the salivary gland, such as β-d-glucosidase, β-d-galactosidase, and lactase floricin hydrolase (LPH), become involved in glycosylated cyanidin metabolite production, such as PCA and aldehyde phloroglucinol (PGA) [21].

Approximately 69% of these anthocyanins are metabolized in the gastrointestinal tract within 4 h of food intake [46]. In a further study, where an oral dose of 50 mg/kg was administered to mice, PCA reached a plasma peak of 73.6 μM in just 5 min. This finding underlines the remarkable rapidity of PCA absorption, with a half-life of 2.9 min [51].

### 3.1. Stomach

In the acidic environment of the stomach, anthocyanins are absorbed at around 10–20% and actively assimilated as primary metabolites via gastric cells. Thereafter, in the liver, a portion of these anthocyanins undergo metabolic processes such as methylation, sulfonation, and glucuronidation. The metabolites generated by these reactions are transported to the intestine via the biliary system [52]. The most relevant phase II metabolic pathways for polyphenols are glucuronidation and sulphation. These metabolic processes significantly impact polyphenols’ physiological characteristics, including solubility, intestinal absorption, tissue distribution, and elimination [53].

### 3.2. Intestines

**Figure 3:**
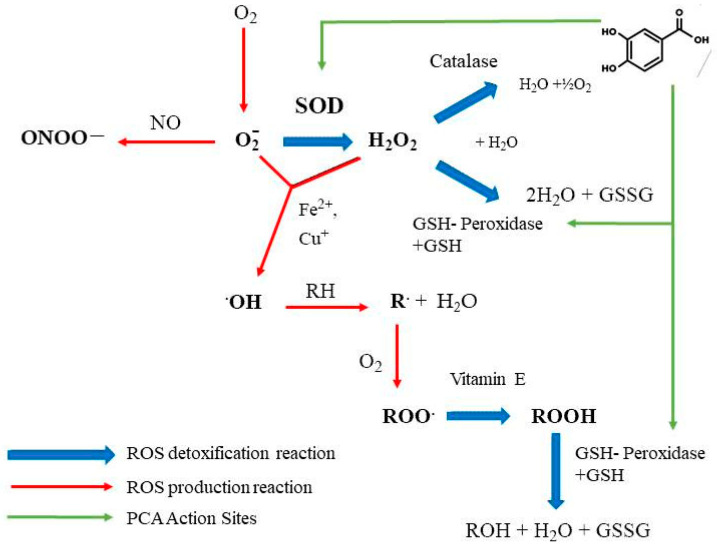
Regulatory action of PCA (Protocatechuic acid) on the oxidation–reduction balance. It noticeably promotes the activity of enzymes GSH-PX and SOD, which act as free radical scavenging enzymes in the cytoplasm.

The intestinal microbiota have high hydrolytic potential and can cleave rings. They play a crucial role in the biological transformation and metabolism of original polyphenolic structures into lower molecular weight metabolites. This process leads to the identification of various anthocyanin degradation products, including vanillic acid, phloroglucinol, and protocatechuic acid [54,55]. Two mechanisms have been proposed for anthocyanin absorption in the intestine. One mechanism is passive diffusion through epithelial cell membranes, as anthocyanin aglycones are highly hydrophobic. The other mechanism involves their passage into the bloodstream as bioavailable anthocyanin products, such as B-ring-derived hydroxybenzoic acids and cyanidin 3-O-glucoside-derived protocatechuic acid [52]. Vitaglione et al. reported that the major metabolite of cyanidin-3-glucoside (Cy-3-glc) in humans is PCA (Figure 3), accounting for an additional 44.4% of the amount ingested into the bloodstream after 6 h [46]. Therefore, it is worth noting that PCA concentration in the body does not correlate proportionally with the amount ingested [4,18,19,45,46].

The large intestine represents the final stage of the digestive process, where a significant portion of anthocyanins remains unchanged until reaching the colon. At this point, the microbial flora continues to transform anthocyanins into simple phenolic acids, such as PCA, increasing their bio-accessibility and contributing to their antioxidant activity. These metabolites are absorbed by colon cells and enter the bloodstream, where they exert their biological effects. In the metabolic process of anthocyanins by microorganisms in the cecum, mainly phenolic acids such as PCA, ferulic acid, gallic acid, syringic acid, p-coumaric acid, and vanillic acid are produced (Figure 2) [48].

After absorption by enterocytes, aglycones travel through the portal vein to the liver. In this organ, they undergo further conjugation, known as phase II drug metabolism, and are transformed into O-glucuronides and O-sulphates. Subsequently, a variable proportion of these phenolic conjugates is eliminated via bile and returned to the small intestine, where they undergo further metabolic cycling. Finally, the resulting phenolic conjugates (O-glucuronides/O-sulphates), such as PCA, are transported into the bloodstream via plasma proteins, where they demonstrate their biological effects before being excreted in the urine [13].

Unabsorbed metabolites are excreted in the feces. These conjugation processes are highly efficient and, as a result, free aglycones are often absent or present in low concentrations in plasma after nutritional doses [55].

It is worth considering that the absorption, distribution, metabolism, and elimination processes of PCA in the human body may differ significantly from those observed in animal models. PCA (0.5 and 5 μg/mL) is known to remain stable in human plasma for 24 h but is rapidly broken down with a half-life of 90 and 314 min in mouse plasma [46].

Regarding PCA absorption in healthy rats, the duodenum has been identified as the main site of absorption, which may occur via passive diffusion. Once absorbed, PCA is distributed to vital organs such as the heart, brain, liver, kidneys, and lungs and is detectable in rat and human blood, feces, and urine [19,46].

A 2007 study by Vitaglione et al. [46] demonstrated a significant increase in blood protocatechuic acid (PCA) concentration following orange juice consumption. The peak concentration (Cmax) reached 492 ± 62 nmol/L at 2 h (tmax) with levels returning near baseline after 6 h. This rapid rise in PCA, observed within minutes of ingestion, has potential physiological and nutritional significance. The presence of PCA in the blood may explain the acute increase in plasma antioxidant activity [46].

The absence of PCA in 24-h urine samples suggests that it may transform and/or bind to other compounds (e.g., serum albumin) [46]. PCA has also been detected in feces with maximum recoveries of 360.9 ± 278.1 µg, 30.0 ± 27.7 µg (PCA-3-sulphate), and 23.0 ± 18.1 µg (PCA-4-sulphate) 6–24 h after ingestion of 500 mg of anthocyanin [45].

## 4. Antioxidant Effects of PCA

Oxidative stress can be defined as the imbalance between the presence of reactive oxygen/nitrogen species (ROS/RNS) and the body’s ability to counteract their actions through the antioxidant protection system. This imbalance is reflected in an increase in ROS/RNS and a decrease in the ability of antioxidant protection, resulting in a deficient capacity of endogenous systems to defend against oxidative attacks directed at biomolecular targets that promote the incidence of diseases and shorten the lifespan [56,57].

The Reactive Oxygen Species (ROS) pool encompasses a variety of molecules such as superoxide anion, hydroxyl radical, singlet oxygen, peroxyl radical, alkoxyl radical, peroxynitrite, hypochlorous acid, and ozone. Reactive nitrogen, iron, copper, and sulfur species are also found [57]. Although some ROS, such as superoxide anion and hydroxyl radicals, are classified as free radicals due to the presence of unpaired electrons, others, such as hydrogen peroxide (H_2_O_2_), do not have unpaired electrons and exhibit remarkable reactivity [58].

Cancer cells are characterized by higher amounts of ROS than healthy cells. They are responsible for maintaining the cancer phenotype as they constitute signaling molecules involved in regulating cell proliferation, apoptosis, and gene expression through the activation of transcription factors [59,60].

High levels of ROS can be explained by the imbalance between oxidants and antioxidants in cancer cells and glycolysis, even in the presence of oxygen. It can also be explained by pyruvate oxidation in mitochondria, known as the Warburg effect. Hypoxia in the tumor microenvironment arises from an imbalance between oxygen supply and consumption, driven by uncontrolled cell proliferation, altered metabolism, and abnormal tumor vasculature. Cancer cells have developed mechanisms to protect themselves from this intrinsic oxidative stress, upregulating survival molecules and their antioxidant defense system to maintain redox balance. For example, nuclear factor erythroid 2-related factor 2 (Nrf2), a transcription factor in the first line of antioxidant defense against oxidative stress, is often upregulated in cancer cells and favors their proliferation [61].

Low molecular weight antioxidants are essential protective agents that mitigate oxidative damage in the human body, especially when internal enzymatic mechanisms fail or are not efficient [62]. Their crucial role contributes to strengthening the immune system and decreasing the incidence of cardiovascular diseases, neurodegenerative diseases, and cancer [63]. Their actions range from reducing oxygen concentration to neutralizing singlet oxygen, preventing the initiation of the oxidative chain by scavenging initial radicals such as hydroxyl radicals, chelating catalytic metal ions, transforming initial oxidation products into non-radical species, and interrupting the chain reaction to prevent the continuous extraction of hydrogen from substrates [12].

When antioxidants fail to neutralize free radicals, damage to lipids, proteins, and genetic material occurs, leading to a variety of effects. Damage to genetic material increases the risk of tumors while cell deterioration and death, resulting from the impact on lipids and proteins, are linked to aging and an increased risk of degenerative diseases [63].

The antioxidant activity of anthocyanins is noted to be 50 times greater than vitamin C and 20 times greater than vitamin E [64]. Their ability to reduce reactive oxygen species (ROS) has been shown to decrease the activation of ROS-induced anti-apoptotic pathways in cancer cells [65].

Specifically, protocatechuic acid (PCA) plays a key role in prevention, mainly due to its outstanding antioxidant activity. They inhibit free radicals, positively activate antioxidant enzymes, and influence the phase 1 and 2 metabolism of specific carcinogens. In addition, PCA may block specific carcinogen binding sites to DNA, preventing the formation of adducts that could lead to mutations and neoplastic transformation [18].

The antioxidant potential of PCA is manifested by increasing endogenous antioxidant enzyme activity, such as glutathione peroxidase (GSH-PX) and superoxide dismutase (SOD) (Figure 3). Furthermore, PCA is considered a perfect scavenger of peroxyl radicals in aqueous environments and a relatively effective antiradical protectant in lipid environments. Additionally, it can attenuate the activity of enzymes such as xanthine oxidase (XOD) and NADPH oxidase (NOX), as well as malondialdehyde (MDA) concentrations (Figure 3) [2].

Protocatechuic acid is known to have remarkable antioxidant capacity by activating the transcription factor Nrf2. In macrophage cells, PCA induces JNK-mediated phosphorylation of Nrf2, which significantly increases the antioxidant enzymes glutathione peroxidase and glutathione reductase. This phenomenon is not only accompanied by a temporary expression of Nrf2 but also by a massive translocation to the cell nucleus. Stabilization of Nrf2 by PCA through post-translational modifications such as phosphorylation suggests a key mechanism for enhancing cellular antioxidant activity [66].

On the other hand, Gao L. et al. [67]. showed that PCA could protect against cisplatin-induced acute kidney injury by reducing oxidative stress and renal inflammation without compromising the drug’s anti-tumor activity, as it suppresses NADPH oxidases, including Nox2 and Nox4, in a dose-dependent manner [67].

## 5. Chemopreventive Capacity of PCA

Cancer chemoprevention encompasses the use of natural, synthetic, biological or chemical substances for the purpose of inhibiting, blocking, suppressing or preventing the progression of cancer to an invasive tumor stage or complete metastasis. Accordingly, chemopreventive agents are categorized as “blocking agents” when their mechanism of action is to inhibit the initiation of tumorigenesis, and as ‘suppressive agents’ when their function is to neutralize tumor promotion or progression [51].

The chemopreventive properties of protocatechuic acid (PCA) encompass its antioxidant effects, ability to chelate metals (especially in the context of ferroptosis), its induction to cell death action (including apoptosis, pyroptosis, and necroptosis), as well as its anti-inflammatory properties and positive regulatory effects on p53 protein [51]. In addition to demonstrating anti-proliferative, anti-angiogenic, and antineoplastic effects on different cell lines both in vivo and in vitro (Table 2), its ability to prevent, inhibit, or reverse tumor formation is highlighted by its interference with the three fundamental stages of chemically induced carcinogenesis [9,10].

At low doses, PCA’s chemopreventive properties are due to its antioxidant capacity [68]. It may also inhibit hepatic P450 activities and suppress the proliferation of malignant cells [69,70]. PCA may, however, exhibit carcinogenic activity because it alters cellular redox balance, decreasing intracellular glutathione and affecting the detoxification of toxins, including ultimate carcinogens [71].

**Table 2:** Molecular basis of PCA action in various cancers in different studies.

Type of Cancer	Cell Line or Animal Used	Activity	Molecular Mechanism	References
Colon Cancer	CaCo-2 cells	Prooxidant, Proapoptotic	Modulation of redox balance and inhibition of the HO-1 system leading to p21 activation.	[72]
Oral Squamous Cell Carcinoma	BALB/CHSC-3 and CAL-27 mice	Apoptosis, Antioxidant	Inhibition of Sb9, Activation of JNK/p3 signaling pathway. Reduced BMI1 and CD44 expression. Decreased ROS excess. Increased SOD and NRF2 expression.	[73]
Esophageal Cancer	Male F-344 rats	Antineoplastic, Antiangiogenic, Anti-inflammatory	Inhibition of tumorigenesis and inflammatory signaling. Induction of PTX3 expression.	[74]
Liver Cancer	HepG2 hepatocellular carcinoma cells	Apoptosis	Induction of JNK-dependent hepatocellular carcinoma cell death.	[67]
Renal Carcinoma	HK2 cells treated with cisplatin	Cytoprotective, Antitumoral	Suppression of cisplatin-induced cell death by suppressing NAPDH oxidases, including Nox2 and Nox4. ↓ ROS.	[75]
Leukemia	HL-60 cells	Apoptosis, Cell cycle arrest	Reduction of Rb phosphorylation. ↓ Bcl-2, ↑ Bax.	[76]
Melanoma	B16F10 and SK-MEL-28 cells	Antimelanogenic	Suppression of α-MSH-induced MITF transcription through negative regulation of AMPc-mediated CREB activation.	[77]
Colorectal Cancer	Human NK cells, Apc Min/+ mice	Cytoprotective, Chemopreventive, Antitumoral	Decrease in inflammatory markers COX-2 and PGE2. Improved expression of IFN-γ and SMAD4 in cultured primary NK cells.	[78]
Human Colon Cancer	WiDr (ATCC CCL-218) and Chang (ATCC CCL-13) cells	Apoptosis, Cell cycle arrest, Pyroptosis	Intrinsic apoptosis by positive regulation of p53, Bax, and caspase-9. Modulation of caspase-8 through the extrinsic pathway. Positive regulation of caspase-1 and -7.	[79]
Hepatocellular Carcinoma	Wistar rats	Apoptosis, Cell cycle arrest	Cytochrome P450 reductase activity and glutathione S-transferase induction. ↓ TNF-α and IL-1β. ↓ Cyclin CDK1. ↑ p53 and Bad, ↓ Bcl-xl.	[80]
Lung Cancer	A549 and H1299 human lung cancer cells	Anticancer	Suppression of fibronectin, vimentin, N-cadherin, MMP-9, MMP-2, twist, and snail. ↑ Epithelial markers E-cadherin and Occludin levels. ↓ Migratory and invasive potential of tumor cells by reversing epithelial-to-mesenchymal transition (EMT). ↓ PI3K/Akt/mTOR activation.	[81]
Lung Cancer	A549, H3255, and Calu-6 cells	Apoptosis, Anticancer	↑ Caspase-3 and Bax, ↓ Bcl-2. Suppressed FAK, NF-κB, and MAPK pathways. ↓ VEGF, fibronectin, bFGF, MMP-2, and MMP-9.	[82]
Gastric Carcinoma	AGS cells	Apoptosis, Repression of Migration, Decreased Matrix Degradation, Inhibition of Metastasis	↓ Ras/Akt/NF-κB. ↓ PI3K, ↑ p53, and the p38 MAPK/FasL pathway.	[83]
Gastric Carcinoma	AGS, MKN45, HepG2, Hep3B	Apoptosis	Activation of JNK/p38 MAPK, both Fas/FasL and p53/Bax apoptotic signaling pathways.	[10]

Up-regulation (↑) and down-regulation (↓).

## 6. Inhibition of Tumors and Metastasis

The conversion of a normal cell to a malignant cell involves the influence of various genomic, genetic, epigenomic, transcriptomic, and proteomic factors. This complex interplay occurs in the tumor microenvironment, where different cell types play a crucial role. Once tumor cells cross the tumor stroma, they can spread into the circulation and colonize distant organs. This process is influenced by the premetastatic niche and the phenomenon of organotropism [84].

The importance of epithelial–mesenchymal transition (EMT) has increased with the growing recognition that this transformation, where cells lose their epithelial identity to acquire mesenchymal characteristics, plays a prominent role in both primary tumor formation and metastatic processes. In addition, various conditions and factors present at tumor margins, such as hypoxia and cytokines secreted by stromal cells, can clearly induce EMT, thus enhancing cancer cells’ invasiveness [3,84].

**Figure 4:**
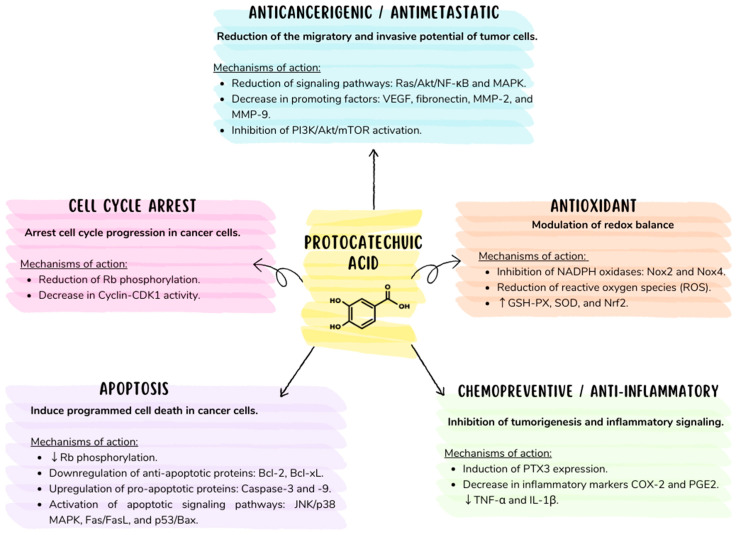
Proposed diagram of PCA’s most relevant biological action mechanisms: (1) apoptosis, (2) anticancer/antimetastatic, (3) cell cycle arrest, (4) chemopreventive/anti-inflammatory, and (5) antioxidant. Up-regulation (↑) and down-regulation (↓).

Degradation of basement membranes and stromal extracellular matrix (ECM) is a critical step in tumor invasion and metastasis formation. The matrix metalloproteinase (MMP) family is responsible for ECM degradation, with MMP-2 and MMP-9 being relevant to native type IV and V collagen, fibronectin, and elastin degradation. MMP gene expression is mainly regulated at the transcriptional level through pathways such as activator protein 1 (AP-1), nuclear factor κB (NF-κB) mediated mitogen-activated protein kinase (MAPK), phosphatidylinositol 3-kinase (PI3K)/protein kinase B (PKB, known as Akt), and at the post-transcriptional level, together with their regulation at the protein level by activators, inhibitors, and cellular localization. Thus, MMPs and their regulatory pathways are potential targets for anticancer drugs and chemotherapeutic agents. In studies on the AGS cell line, PCA exhibited anti-metastatic effects by negatively regulating the Ras/Akt/NF-kB pathway, leading to the subsequent inhibition of MMP-2 secretion and inability to metastasize [83] (Figure 4).

PCA can also modulate TGFβ-induced expression of mesenchymal and epithelial markers, reversing tumor cells’ migratory and invasive potential by restoring the epithelial state and slowing EMT. In lung cancer cells, PCA suppresses the EMT process by inhibiting the activation of the PI3K/Akt/mTOR signaling pathway [81].

A study by Dong et al. (2022) [78] highlighted PCA’s efficacy in reducing neutrophils in esophageal papilloma tissue, modifying immune cell mobilization, and preventing inflammation. In addition, it was observed to positively regulate the expression of the promoter of pentraxin 3 (PTX3), a silenced gene in esophageal cancer that inhibits angiogenesis and tumorigenesis when activated [78].

Moreover, a concentration of 500 ppm of PCA in the diet sufficiently suppresses carcinogenesis in several organs, such as the tongue, stomach, colon, liver, and bladder of rats [18]. Similarly, protective effects were observed when 2000 ppm PCA was administered in the diet, particularly in tongue cancer progression, suggesting that concentrations below 2000 ppm may effectively inhibit all stages of carcinogenesis (initiation, promotion, and progression) [85].

These findings suggest PCA’s potential as a valuable compound for cancer prevention. However, the amounts consumed by humans in their daily diet may be considerably lower than effective doses observed in research studies [18].

## 7. Mechanism of Apoptotic Action in PCA

Overall, PCA has shown remarkable ability to positively regulate proapoptotic proteins such as Bid, Bax, and the caspase-mediated death-signaling cascade. Conversely, it has interfered with the Bcl-2 family’s activity on anti-apoptotic proteins, generating an environment conducive to apoptosis induction in cancer cells [8] (Figure 4).

In a study made by Tsui-Hwa Tseng et al. [76], protocatechuic acid was effective as an inhibitor of human promyelocytic leukemia cell survival (HL-60) depending on concentration and exposure time. This effect was achieved by reducing the phosphorylation of the retinoblastoma (Rb) protein and decreasing Bcl-2 expression while increasing Bax expression, a fundamental protein in regulating apoptotic processes [76]. On the other hand, research conducted on the HEL cell line by Zambonin et al. reaffirmed that PCA exhibited apoptosis-promoting effects in leukemic cells. In healthy cells, it manifested apoptosis-inhibiting effects [65].

Furthermore, PCA triggers cell death and apoptosis in HepG2 cells at concentrations of 100 μmol/L and human gastric carcinoma cells (AGS) at concentrations of 1–8 mM by activating JNK/p38 signaling [75]. On the other hand, PCA plays a crucial role in inhibiting osteoclast differentiation by reducing oxidative stress levels and associated genes through key transcription factors such as NF-kB and Nrf2. It also promotes apoptosis in mature osteoclasts by activating caspases [86].

In another investigation, it was observed that PCA induced programmed cell death through different pathways depending on the dose used. At lower concentrations (1–10 µg/mL), it stimulated the intrinsic apoptosis pathway by positively regulating the upregulation of p53, Bax, and caspase-9. At the same dose level, it also activated the extrinsic pathway by modulating caspase-8. On the other hand, PCA showed positive regulation of caspase-1 and caspase-7 at higher doses (25–50 µg/mL), suggesting its possible involvement in proinflammatory cell death [79].

## 8. Perspectives

A multitude of studies have shed light on the therapeutic potential of protocatechuic acid (PCA) in cancer treatment. This multifaceted compound exerts its influence on a diverse array of physiological processes, regulating their proper functioning. While the precise molecular mechanisms by which PCA disrupts specific pathways leading to cell death remain to be fully elucidated, the need for further in vivo investigations to determine an optimal PCA concentration is evident. Research consistently demonstrates the dose dependent nature of PCA. Authors like Nakamura et al. (2000) caution that excessive consumption of PCA may have an adverse, carcinogenic effect due to its ability to disrupt the cellular redox balance.

Other authors mention that in high concentrations, such as 800 mg/Kg, peritoneally and 3.5 mg/Kg intravenously in mice, it can be lethal [9], however a concentration of 2000 mg/kg administered in the diet can exert a suppressive effect on cancer of the tongue, stomach, colon, liver, and bladder in rats [85]. Other studies mention that the aqueous extract of PCA administered daily with a concentration of 500 mg/kg decreases oxidative stress and prevents cellular degeneration and necrosis of kidney tissues, reducing serum levels of urea and creatinine and significantly increasing levels of GSH and SOD [51]. Other investigations propose a concentration between 50 and 150 mg/Kg having significant non-toxic results [51].

Considering these studies, it is necessary to carry out more in vivo research to de-termine an adequate dose of consumption and also the route of administration, a key factor for the beneficial effect of PCA and which can be a tool for cancer prevention. This constant challenge is positioned as a crucial area for the medical and scientific community. Despite this, protocatechuic acid (PCA) is seen as an extremely promising compound in the management of various diseases, with potential chemopreventive for relevant clinical applications in the prevention of neoplastic diseases.

## 9. Conclusions

Extensive research highlights the chemoprevention potential of protocatechuic acid (PCA) and its diverse properties, including antioxidant, anti-inflammatory, antiapoptotic, and chemoprotective effects. These features hold promise for mitigating inflammatory processes and managing oxidative stress, which significantly contributes to various health concerns. However, the precise molecular mechanisms by which PCA influences cell death pathways remain unclear, and further research is crucial.

PCA’s potential represents fertile ground for future exploration, both in laboratory and clinical settings. These investigations are essential to fully understand PCA’s capabilities and explore its potential synergistic effects with chemotherapeutic agents. Moreover, PCA’s potential as a complementary approach to cancer prevention and treatment warrants further investigation. This pursuit toward a deeper understanding of PCA’s benefits paves the way for the development of more effective and personalized strategies in the fight against cancer.

## Figures and Tables

**Table 1:** Concentration of PCA in different plant-based foods (flowers and fruits).

No.	Flower or Fruit	Concentration (mg/kg)	Reference
1	*Solanum tuberosum* L. (potato: peel).	2560	[22]
2	*Amaranthus caudatus* L. (amaranth: seeds, leaves, and flowers).	0.0136	[23]
3	*Cnidoscolus chayamansa* (Mill.) I.M.Johnst (chaya: leaves).	242 ± 0.001	[24]
4	*Hibiscus sabdariffa* L. var. *Alma blanca* (hibiscus: leaves, root, stem, capsule, and whole and ground seeds).	86.2	[25]
5	*Hibiscus sabdariffa* L. var. *Chiautla* (hibiscus: leaves, root, stem, capsule, and whole and ground seeds).	81	[25]
6	*Hibiscus sabdariffa* L. var. *Huajicori* (hibiscus: leaves, root, stem, capsule, and whole and ground seeds).	1397	[25]
7	*Hibiscus sabdariffa* L. var. *Tecoanapa* (hibiscus: leaves, root, stem, capsule, and whole and ground seeds).	135.1	[25]
8	*Rubus idaeus* L. (Raspberry).	100	[26]
9	*Oleo europaea* L. (Olive: olive oil).	0.22	[27]
10	*Cicer arietinum* L. (chickpea: sprouted, roasted, pressure-cooked, and microwave-heated seeds).	514.2	[28]
11	*Mangifera indica* L. (mango: mango pulp).	7.7–68.3	[29]
12	Oryza sativa L. (rice: whole and soaked grain).	23.2–1043	[30]
13	*Fagopyrum esculentum* Moench (buckwheat: whole grain and husk).	6.61–24.5	[31]
14	*Pisum sativum* L. (green pea: green pea flour).	1.26–11.38	[31]
15	*Vicia faba* L. (broad bean: broad bean flour).	0.61–2.42	[31]
16	*Cannabis sativa* L. ((hemp: hemp flour).	5.63–22.06	[31]
17	*Lupinus albus* L. (lupin: lupin flour).	0.15 ± 0.02	[31]
18	*Triticum aestivum* L. (common wheat: wheat flour).	0.07–0.11	[31]
19	*Lens culinaris* Medik (lentils: whole dried seeds).	20.28–37.72	[32]
20	*Phaseolus vulgaris* L.(common bean: ground whole grain).	95.34–253.42	[33]
21	*Theobroma cacao* L. (Cocoa: cocoa bean).	197.9–385.3	[34]
22	*Allium cepa* L. (onion: outer layers of the onion).	1027	[35]
23	*Musa × paradisiaca* L. (Banana: banana pulp).	340	[36]
24	*Ribes rubrum* L. (Red currant: whole freeze-dried fruit).	137.6–464.8	[37]
25	*Hypericum perforatum* L. (St. John’s Wort: aerial parts).	761.67	[38]
26	*Olea europaea* L. (Olive: olive leaves).	176.08	[38]
27	*Hibiscus sabdariffa* L. (*Hs*, *Roselle*) (Hibiscus: dried flowers).	94.1	[39]
28	*Cynomorium songaricum Rupr*. (Chinese herb: lyophilized herb).	148	[40]
29	*Prunus amygdalus* Batsch (almonds: almond shells).	66.67	[41]
30	*Sechium edule* (Jacq.) Sw. (chayote 30 Gy SC-*S. compositum)*	1910	[42]
31	*Sechium edule* (Jacq.) Sw. (chayote 10 Gy H387b)	1050	[42]

## Data Availability

The data presented in this study are available in article.
